# Ratiometric Electrochemical Detection of Interleukin-6 Using Electropolymerized Methylene Blue and a Multi-Walled Carbon-Nanotube-Modified Screen-Printed Carbon Electrode

**DOI:** 10.3390/bios14100457

**Published:** 2024-09-25

**Authors:** Zhuo Liu, Fengyu Liu, Chaofan Wang, Hongjuan Li, Yongqian Xu, Shiguo Sun

**Affiliations:** 1 College of Chemistry & Pharmacy, Northwest A&F University, Xianyang 712100, China; liuzhuo@nwafu.edu.cn (Z.L.); kmln0201@nwafu.edu.cn (C.W.); hongjuanli@nwafu.edu.cn (H.L.); xuyq@nwsuaf.edu.cn (Y.X.); 2 School of Chemistry, Dalian University of Technology, No. 2 Linggong Road, Ganjingzi District, Dalian 116023, China; 3 Shenzhen Research Institute, Northwest A&F University, Shenzhen 518000, China

**Keywords:** electropolymerized methylene blue, multi-walled carbon nanotubes, electrochemical sensor, screen-printed carbon electrode

## Abstract

Herein, we report a ratio-based electrochemical biosensor for the detection of interleukin-6 (IL-6). We electropolymerized methylene blue (MB) on the surface of screen-printed carbon electrodes; introduced an internal reference signal probe; modified the carboxylate multi-walled carbon nanotubes on the electrode surface to increase the electrochemically active area; and finally linked the amino-modified IL-6 aptamer to the electrode surface through the Schiff base reaction, with bovine serum albumin (BSA) added to mask non-specific adsorption. After adding IL-6 to the samples, the signal of I_MB_ remained almost unchanged, while the signal of I[Fe(CN)_6_]^3−/4−^ decreased with increasing IL-6 concentration. Thus, a novel ratiometric electrochemical sensor with a linear range of 0.001~1000.0 ng/mL and a low detection limit of 0.54 pg/mL was successfully developed. The sensor had high repeatability, stability, sensitivity, and practicability. It provides a new method for constructing proportional electrochemical sensors and detecting IL-6.

## 1. Introduction

Cytokines are important signaling molecules that have been playing a crucial role in the human immune system. They are crucial for maintaining the physiological balance of the human body and responding to various challenges. IL6 is a 21–28 KDa glycosylated protein that plays a crucial role in the regulation of homeostasis in humans via proinflammatory cytokine and anti-inflammatory cytokines [1,2,3,4]. It is typically produced in response to tissue damage and infection, and elevated IL-6 levels are associated with numerous diseases. The physiological concentration of IL-6 in normal human serum is relatively low, about 1–5 pg/mL, but in various inflammatory conditions (such as rheumatoid arthritis and meningitis), IL-6 levels increase rapidly [5,6,7]. Therefore, the rapid and highly specific detection of IL-6 is crucial for the diagnosis and treatment of early-stage inflammation.

There are several methods available to detect IL-6, including colorimetric analysis, flow cytometry, enzyme-linked immunosorbent assay, polymerase chain reaction, and chemiluminescence immunoassay [4,8,9,10]. However, these techniques are challenging to use for rapid and real-time detection due to their complexity, high cost, and time-consuming nature [11]. Electrochemical biosensors enable rapid response, high sensitivity, and real-time monitoring by converting chemical reactions into measurable electrical signals [12,13,14]. For example, Ting et al. designed a label-free electrochemical sensor that can detect IL-6 very sensitively [15]. Similarly, Huang et al. developed a portable, sensitive electrochemical biosensor with a smartphone that is superior to enzyme-linked immunosorbent assay (ELISA) in detecting serum samples [16].

Although there have been some reports of electrochemical detection of IL-6, these studies are based on a single-signal detection mode, which is susceptible to interference from many factors [17,18], such as environmental change, instrument interference, and electrode passivation [19,20]. To make up for these shortcomings, proportional electrochemical sensors have received extensive attention. By measuring the ratio of peak current between two electrochemical signals of different reduction potentials (target analyte signal and reference signal), it achieves effective self-calibration, significantly improving the repeatability, stability, and reliability of the sensor [21,22]. Previously, our team used ferrocene as an internal standard substance, coated it on the surface of a glassy carbon electrode, and achieved simultaneous detection of glycyrrhizin and isoliquiritigenin using molecular imprinting technology [23].

Often to add a ratio measurement signal, electrochemical probes like methylene blue (MB), ferrocene (Fc), and thionine (Thi) are frequently chosen as reference probes. Among them, MB is often utilized as a ratio signal due to its outstanding stability, non-interference with or response to the material under test, generation of a strong electrical signal, and the distance between its peak position and the response peak of the material under test being capable of avoiding mutual interference [21]. However, poly(methylene blue) (pMB) is known to have poor electrical conductivity. Therefore, we introduced carbon nanotubes (CNTs), which offer good conductivity, high specific surface area, low cost, outstanding chemical and mechanical stability, and facile surface modification. Additionally, the introduction of CNTs provides more sites for aptamer modification [24].

In this paper, an aptamer-based electrochemical sensor was developed for detecting IL-6 protein in serum samples. An internal reference was introduced by polymerizing MB on a screen-printed carbon electrode (SPCE). Subsequently, carboxylated multi-wall carbon nanotubes (MWCNTs) were modified on the electrode surface to increase the electrochemically active electrode area. A Schiff base reaction between the carboxyl group and the amino group on the aptamer was then used to link the IL-6 aptamer to the electrode surface. Finally, bovine serum albumin (BSA) was used to mask non-specific adsorption, and the sensor was further modified. Differential pulse voltammetry (DPV) was used to specifically detect IL-6. When the target protein was present, the peak current of MB did not change due to its role as an internal reference. However, the current of I[Fe(CN)_6_]^3−/4−^ decreased because the aptamer adsorbed the protein. Consequently, the signal ratio of I[Fe(CN)_6_]^3−/4−^ to I_MB_ was self-calibrated to reflect the specific concentration of the target molecule in the range of 0.001–1000.0 ng/mL, and the detection limit was 0.54 pg/mL. Finally, the electrochemical sensor was applied to serum labeling analysis with good results. In summary, this paper aims to provide a new strategy for the diagnosis of other clinical biomolecules by realizing the quantitative detection of IL-6.

## 2. Materials and Methods

### 2.1. Materials

MB, sodium chloride, potassium chloride, disodium phosphate, sodium dihydrogen phosphate, sodium hydroxide, and n-hydroxysuccinimide were purchased from Macklin (Shanghai, China). MWCNTs were purchased from XFNANO (Nanjing, China). Chitosan, glacial acetic acid, nitric acid, and sulfuric acid were purchased from Xilong Scientific Co., Ltd. (Shantou, China). 2-(N-morpholine) ethanesulfonic acid monohydrate (MES), 1-(3-dimethylaminopropyl)-3-ethylcarbodiimide (EDC), BSA, and serum albumin (HSA) were purchased from Aladdin (Shanghai, China). IL-6 and IL-8 proteins were purchased from Sigma-Aldrich (Shanghai, China), and IL-6 adaptor 5′-GGTGGCAGGAGGACTATTTATTTGCTTTTCT-NH_2_-3′ was commissioned by bioengineering (Shanghai, China) Co., Ltd. [25].

### 2.2. Instruments

The electrode modification process was characterized using a scanning electron microscope (SEM, Nano SEM-450, Brno, Czech Republic). Electrochemical measurements were performed at the electrochemical workstation (CHI660E, Shanghai, China) using an SPCE with a diameter of 3 mm (0.071 cm^2^). Ag/AgCl ink is coated on the electrode surface as a reference electrode, and carbon ink as a reverse electrode. And SPCEs were purchased from Weihai Poten Technology Co., Ltd. (Weihai, China). The cyclic voltammetry (CV) parameters were as follows: scan range of −0.2–0.6 V, scan rate of 50 mV/s; electrochemical impedance spectroscopy (EIS) parameters: amplitude of 5 mV, frequency of 0.01–100 kHz, polarization voltage as open circuit voltage; DPV parameters: potential scanning range of −0.2–0.6 V, amplitude of 0.025 V, pulse width of 0.05 s, sampling interval of 0.02 s, pulse time of 0.2 s, settling time of 0 s. All electrochemical measurements were conducted at room temperature.

### 2.3. Preparation of Chitosan-Carboxylic Multi-Wall Carbon Nanotubes

To introduce the carboxyl group into MWCNTs, the carboxylic acid was treated with an oxidizing reagent mixed with strong acid. First, 1 g of MWCNTs was dispersed in a round-bottomed flask filled with 100 mL of mixed acid (V(H_2_SO_4_):V(HNO_3_) = 3:1) solution, mixed uniformly at room temperature for half an hour, and then reflowed at 100 °C for 12 h. After the system was allowed to cool to room temperature, the product was diluted and filtered, rinse with deionized water to precipitate to neutral, then rinsed with ethanol three times, and following this was transferred to a vacuum drying box to dry [26].

Then, 2 mg of chitosan was added to 1 mL of the 1% acetic acid solution to dissolve; subsequently, 0.4 mg of the MWCNTs obtained in the previous step were taken and ultrasonically dispersed in 1 mL of deionized water for 30 min, and then they were mixed with the above-mentioned chitosan acetic acid solution for an ultrasound for 1 h to obtain a uniform dispersion; following this, they were stored in a 4 °C refrigerator for later use.

### 2.4. Preparation of Ratio Electrochemical Sensor

The SPCE was placed in a solution of PBS (0.1 mol/L) containing 1.0 mmol L of MB monomer and subjected to electropolymerization by potentiometric cycling at a pH of 7.2. To this end, 40 potential cycles (−1.0 to +1.2 V) were performed at 100 mV/s. After the electropolymerization was complete, the electrode was cleaned with deionized water and then subjected to 5 potential cycles at 50 mV/s in 0.1 mol/L PBS to remove unreacted monomer MB [27].

Then, the dispersed MWCNTs were fixed on the surface of the SPCE working electrode, and the modified electrode was obtained by drying at room temperature overnight. For details about how to link aptamers, refer to the method of Masoud [28]. Carboxyl groups on MWCNTs were activated by the infusion of a mixture of MES buffers, EDC, and NHS. After 30 min, the electrode was cleaned with deionized water, and the IL-6 aptamer was immediately dropped into the electrode. Because the aptamer is designed as a hairpin structure [25], the exposed amino group is easily coupled to the carboxyl group of MWCNTs. The aptmer-NH_2_-modified electrode was then immersed in 6 μL 0.1% BSA solution for 30 min to block the non-specific adsorption of MWCNTs to other substances and finally stored at 4 °C overnight.

## 3. Results and Discussion

### 3.1. Characterizations

**Figure 1:**
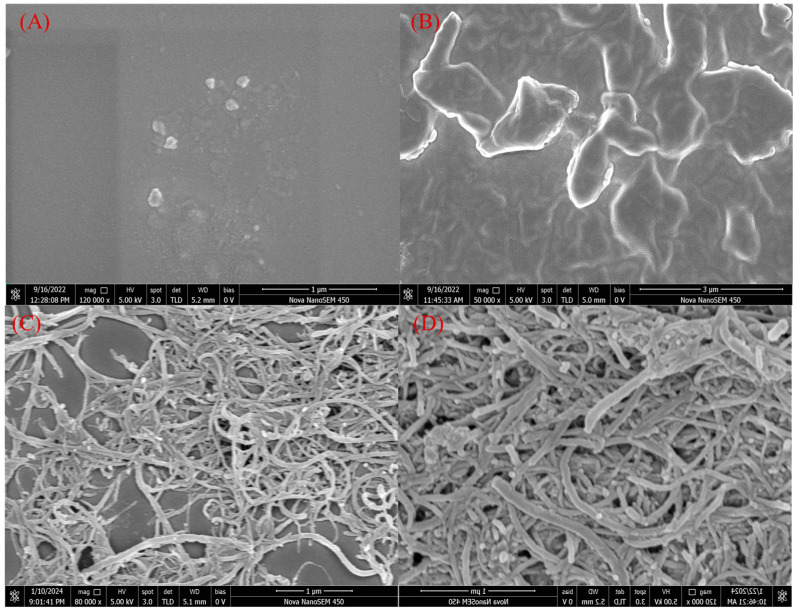
SEM images of (**A**) bare SPCE, (**B**) SPCE modified with pMB, (**C**) MWCNTs, and (**D**) CS/MWCNTs.

The surface of the bare SPCE was black, and due to the absence of electrochemical deposition, the bare SPCE surface only had a carbon film. SEM observation shows that the surface of the SPCE was relatively flat with small particles, which may have been caused by the uneven carbon material during the screen-printing process (Figure 1A). After electrochemical deposition, the surface of the working electrode can be seen as a light red color with the naked eye. Through SEM observation, a layer of pMB film was formed to cover the electrode surface, making the surface more uneven (Figure 1B). The morphology of MWCNTs and CS/MWCNTs was observed by SEM. MWCNTs showed curved thin-walled tubular structures with varying lengths (Figure 1C). By comparing the SEM images of CS/MWCNTs with the scanning electron microscopy images of MWCNTs (Figure 1D), it can be observed that the tubular structures became more opaque and their diameters also increased, indicating that CS successfully coated the surface of MWCNTs [29].

**Figure 2:**
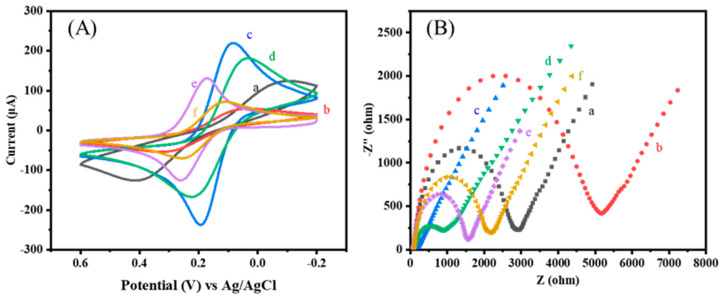
(**A**) CV curves (scan rate 50 mV s^−1^) and (**B**) EIS curves of different treated electrodes in 0.1 M KCl containing 5 mM [Fe(CN)_6_]^3−/4−^: (a) the bare SPCE, (b) SPCE-pMB, (c) SPCE-pMB-CS/MWCNTs, (d) SPCE-pMB-CS/MWCNTs-apt, (e) SPCE-pMB-CS/MWCNTs-apt-BSA, (f) 1 ng/mL-IL-6.

As shown in Figure 2A, naked SPCE exhibited a pair of [Fe(CN)_6_]^3−/4−^ redox peaks when unmodified (a). However, after electrodepositing MB on the surface of the SPCE (b), the redox peaks decreased. This was due to the deposition of a pMB film on the electrode surface, which was less conductive than the original carbon substrate, resulting in reduced current. Subsequently, a layer of CS/MWCNTs was dripped onto the surface of the SPCE. MWCNTs have a high specific surface area, and the electroactive surface area of the SPCE was significantly increased, resulting in a maximum redox peak (c). With the addition of amino-modified ligands, the peak redox current decreased (d), attributed to the weak electrical conductivity of the ligands, which hinders electron transfer between the nanomaterials. After the addition of masking agent BSA, the peak current further decreased. This was due to BSA further reacting with the unbound sites of MWCNTs, hindering current transfer and causing a leading in current (e). Finally, the IL-6 protein bound specifically to the aptamer, which increased the steric resistance of the electrode surface and further decreased the current signal value (f). The above changes in the value of the current signal indicate the successful preparation of the electrochemical sensor [27].

The electrochemical characteristics of the electrode modification process were further analyzed by EIS, and the results were consistent with CV analysis. The low-frequency region of the AC impedance spectrum exhibited a linear curve, while the high-frequency region displayed a semicircular shape. The diameter of this quasi-semicircle represents the charge transfer resistance (Rct), which increases with the diameter of the semicircle in the high-frequency region. Figure 2B illustrates the impedance spectra obtained during various stages of the modification process. In the Nyquist plot, the bare SPCE (a) exhibited relatively high electron transfer resistance. After modification with pMB (b), the semicircle diameter increased, consistent with the poor conductivity of the film. Subsequently, MWCNTs were introduced (c), and due to their excellent conductivity, the semicircle diameter decreased significantly. With the successive modification of the aptamer (d) and BSA (e), the semicircle diameter increased, and Rct correspondingly increased, indicating that the EIS results align with the CV results, further confirming the successful construction of the electrochemical biosensor.

### 3.2. Construction Process and Optimization of Experimental Conditions for the Ratiometric Immunosensor

**Figure 3:**
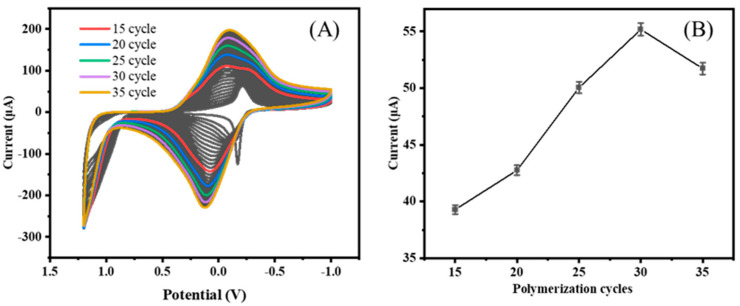
CV of MB (**A**) and the peak current of the modified electrode in potassium ferricyanide solution (**B**).

To achieve optimal performance of the constructed aptamer sensor, the cycle number of MB polymerization cycles, CS/MWCNT loading, aptamer concentration, and IL-6 protein incubation time were optimized using the single-factor variable method. The current signal detected by DPV was also analyzed and interpreted. The method of electro-polymerization was used to prepare pMB film, which is a method that makes it easy to control the thickness and morphology of the film. As is evident from Figure 3, the electrodeposited MB electrode showed quasi-reversible redox in PBS solution, with a distinct redox peak at about −0.1 V, which agrees with the literature [30]. The MB current increased with the number of polymerization cycles, and the polymerization slowed down after the 30th cycle. This is because the electrode surface had completely covered a layer of pMB film, and further increasing the cycle number of polymerization cycles resulted in a decrease in the polymerization rate. At the same time, the electrode showed a decrease in the MB current, and the surface MB was loaded to reach the upper limit. Continuing polymerization will reduce the MB load. Therefore, 30 cycles were selected as the optimal polymerization cycle number for MB.

**Figure 4:**
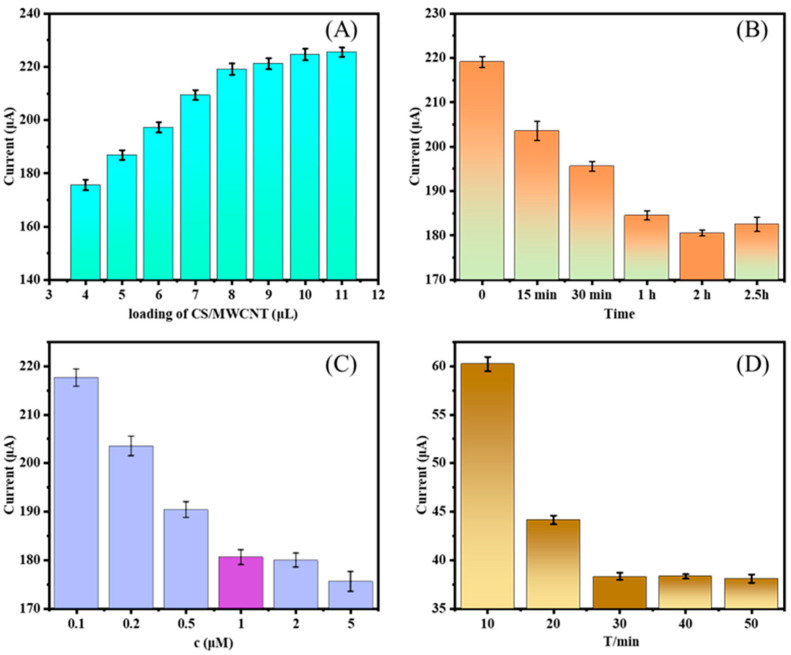
Effects of CS/MWCNTs loading (**A**), aptamer incubation time (**B**) and concentration (**C**), and IL-6 protein incubation time (**D**) on electrical signals (*n* = 3).

As shown in Figure 4A, the effect of CS/MWCNTs loading on the response signal of electrochemical sensors was investigated. A low loading will reduce the enrichment of target molecules, while an excessive loading will cause material waste. As is evident from Figure 4A, the current significantly increased when adding 4–8 μL composite materials dropwise, but after 9 μL, although the current increased, it was very little. The surface material was close to load saturation, so 8 μL material was chosen as the optimal load amount. As shown in Figure 4B, the electrochemical signal response gradually decreased with increasing incubation time, indicating that the MWCNTs modified by amino and carboxyl groups through the aptamer were coupling, reaching the minimum value at 2 h. Therefore, 2 h is the optimal incubation time. The optimization of aptamer concentration helps to obtain the minimum concentration required for the optimal sensing interface, thereby effectively saving costs.

As shown in Figure 4C, the detection electrochemical signal response decreased with the increase in amino ligand (NH_2_-Apt) concentration from 0.1 μM to 5 μM and reached its lowest point at a concentration of 1 μM, indicating that the binding between the ligand and MWCNTs was close to saturation. Subsequent increases in concentration will only cause material waste. Therefore, a NH_2_-Apt concentration of 1 μM was chosen as the optimal condition. The time required for the combination of aptamer and IL-6 is also a crucial step in the preparation of high-performance sensors. The IL-6 incubation process of the electrochemical aptamer sensor in this experiment was carried out under stirring with known standard solutions of different concentrations, as shown in Figure 4D. Within the incubation time of 0–30 min, the current difference significantly decreased, indicating that the aptamer on the modified electrode was specifically bound to IL-6, hindering the redox reaction of potassium ferrocyanide on the electrode surface. When the incubation time was 30 min, the current value tended to stabilize and reach its lowest point. Continuing to increase the incubation time, the current response value no longer showed significant changes. Therefore, the incubation time for IL-6 was chosen as 30 min.

### 3.3. Performance Analysis of the Ratiometric Immunosensor

**Figure 5:**
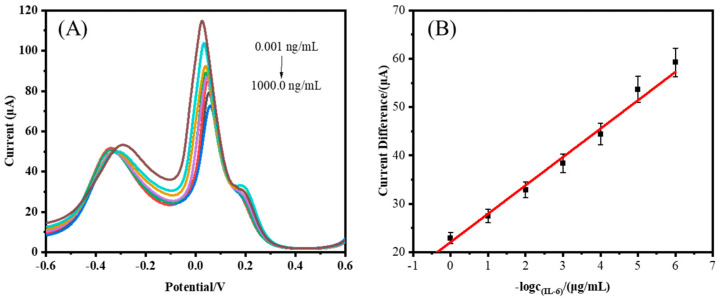
(**A**) DPV plot of the electrochemical sensor at different IL-6 concentrations (From the top to the bottom of the corresponding concentration: 0 ng/mL, 0.001 ng/mL, 0.01 ng/mL, 0.1 ng/mL, 1 ng/mL, 10 ng/mL, 100 ng/mL, 1000 ng/mL.) and (**B**) standard curve of current difference versus negative logarithmic value of IL-6 concentration (*n* = 3).

Figure 5A shows the current response schematic of the electrochemical aptamer sensor in IL-6 protein solutions of different concentrations (0.001~1000.0 ng/mL in the solution). As is evident from Figure 5A, the difference in peak current between [Fe(CN)_6_]^3−/4−^ and MB ranged from 0.001 ng/mL to 1000.0 ng/mL, and it decreased with increasing protein concentration. This was due to the specific binding of IL-6 protein to the aptamer, which increased the steric hindrance on the electrode surface and hindered electron transfer, resulting in the peak current of [Fe(CN)_6_]^3−/4−^ decreasing. However, MB forms a film on the electrode surface and does not undergo significant current changes, so the difference between the two will decrease with increasing protein concentration. According to the calculated Figure 5B, the working curve for detecting the IL-6 protein is shown. Within the range of 0.001 ng/mL to 1000.0 ng/mL, the peak current difference signal of DPV had a good linear relationship with the logarithm of IL-6 concentration. The linear regression equation is ∆I (Fe/MB) = −5.8635lgc − 13.0995 (∆I (Fe/MB) is the peak current of potassium ferrocyanide minus the MB peak current, and c is the IL-6 protein concentration), R^2^ = 0.9827, and the LOD is 0.54 pg/mL. In addition, the electrochemical sensor proposed in our study had a relatively LOD and a wide linear range compared to other reported sensors (Table 1).

**Figure 6:**
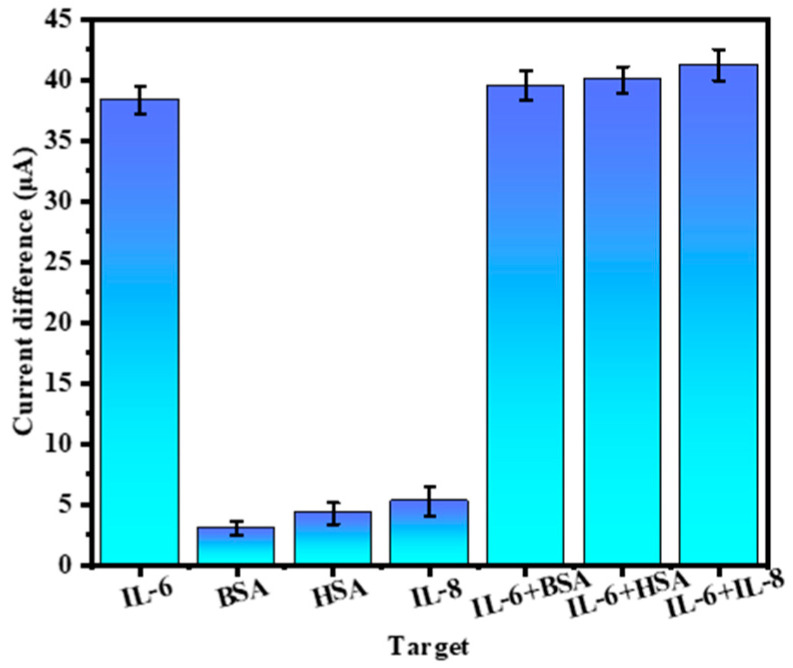
Electrical signal response of IL-6 and other interfering proteins during detection (*n* = 3).

Then, the repeatability, specificity, and stability of the sensor were investigated. Firstly, aiming to investigate the specificity of the prepared electrochemical sensor, 10.0 ng/mL BSA, 10.0 ng/mL HSA, 10.0 ng/mL IL-8, 1.0 ng/mL IL-6, and their mixtures with various proteins were detected under optimal experimental conditions (here, BSA was utilized as a large molecular weight protein, HSA as a common human protein, IL-8 and IL-6 as cytokines; interference proteins and IL-6 were mixed in a concentration ratio of 10:1) [25]. The results are shown in Figure 6, the current difference of IL-6 was significantly higher than that of other proteins, and the mixed protein results also showed that the electrode current difference would significantly decrease when IL-6 was present. The above results show that the electrochemical sensor has good specificity to IL-6 and has certain anti-interference ability.

**Figure 7:**
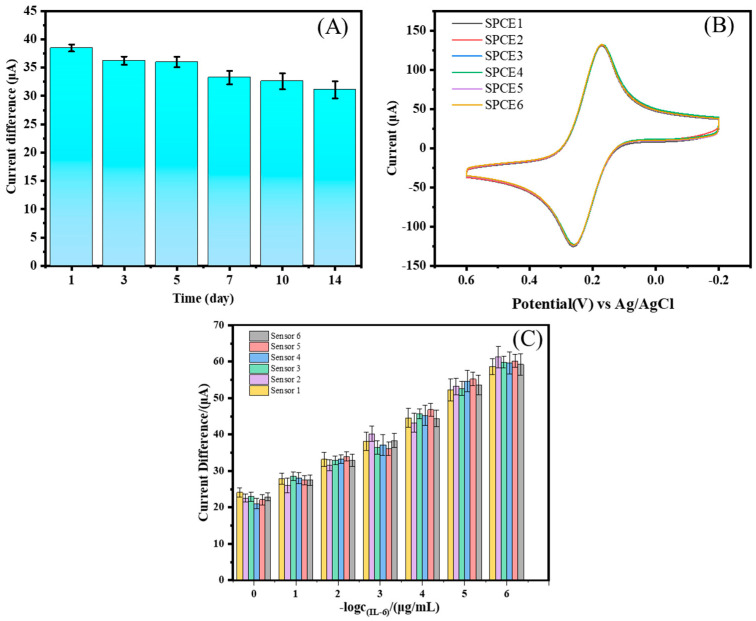
Stability (**A**) and reproducibility (**B**) of the electrochemical sensor. (**C**) The change of current difference when the same batch of sensors detected IL-6 (*n* = 3).

Then, a batch of electrochemical aptamer sensors was prepared under the same conditions. The electrodes were stored at 4 °C and then taken out at different times. An IL-6 protein solution containing 1 ng/mL was used for measurement, and the peak current difference was recorded. The results are shown in Figure 7A. After two weeks, the current response value became 82.1% of the original value, indicating that the sensor can be stored for some time. Similarly, six different SPCE functional electrodes were produced in the same batch to evaluate the reproducibility of the sensor. As shown in Figure 7B, six of the sensors were chosen to check the current changes, with a relative standard deviation (RSD) of 3.7%, indicating that the electrochemical sensor constructed with different electrodes has good reproducibility.

To further explore the repeatability of the sensor, six electrodes were used to detect IL-6, as shown in Figure 7C, and there was not much difference between them. At the same time, a *t*-test was carried out on the six groups of data, and their P values were all greater than 0.95 (Table 2), indicating that there is no significant difference among the six groups of data, which further indicates that the sensor has good repeatability [25]. In summary, the electrochemical sensor has high reproducibility and good stability for the detection of IL-6 protein.

### 3.4. Application of IL-6 Sensor in Human Serum

We detected human serum samples through serum labeling experiments. Different concentrations of IL-6 protein were added to serum samples diluted 10 times for detection. The labeled serum samples were placed on the surface of the working electrode of the prepared electrochemical sensor and incubated for 30 min, and we then cleaned the electrode with PBS and conducted DPV analysis in a [Fe(CN)_6_]^3−/4−^ PBS solution containing 1 mmol/L. The results are shown in Table 3. The recovery rate of IL-6 in serum samples was 94.9%–103.0%, and the relative standard deviation was about 3.0%. The electrochemical sensor had good feasibility for detecting IL-6 in human serum, indicating that it can achieve real-time and rapid detection of human serum samples.

## 4. Conclusions

In summary, we used electropolymerization MB, introduced internal reference signals, and subsequently modified MWCNTs to enhance the electrochemical activity area, as well as detecting the IL-6 protein specifically by modifying the aptamer, wherein the sensor was applied to the spiked detection of IL-6. The detection limit of IL-6 was 5.4 × 10^−4^ ng/mL. In addition, the sensor had ideal selectivity, stability, and repeatability, and it had great potential in practical applications.

## Figures and Tables

**Table 1:** Comparison of different methods for detecting IL-6.

Detection Method	Concentration Range/(ng/mL)	LOD/(ng/mL)	Reference
Homemade instruments	1–200	1	[31]
FRET	1.5–5.9 × 10^−3^	8.2 × 10^−4^	[32]
SERS	0.1–1000	0.05	[33]
EIS	0.01–10	5 × 10^−2^	[34]
DPV	0.01–5	3.5 × 10^−3^	[18]
DPV	0.001–1000	5.4 × 10^−4^	This work

**Table 2:** *t*-test between different electrodes.

	1	2	3	4	5	6
*t*-test/*p*		0.95	0.99	0.99	0.98	0.99

**Table 3:** Determination of IL-6 in human serum with spiked method.

Serum Samples	Added/(pg/mL)	Found/(pg/mL)	Recovery/%	RSD/%
1	5.0	5.152 ± 0.17	103.0	3.29
2	10.0	10.254 ± 0.26	102.5	2.53
3	20.0	18.984 ± 0.68	94.9	3.58
4	50.0	51.024 ± 0.98	102.0	1.92
5	100.0	98.976 ± 2.88	99.0	2.91

## Data Availability

Data are contained within the article.
